# High-throughput microplastic assessment using polarization holographic imaging

**DOI:** 10.1038/s41598-024-52762-5

**Published:** 2024-01-29

**Authors:** Yuxing Li, Yanmin Zhu, Jianqing Huang, Yuen-Wa Ho, James Kar-Hei Fang, Edmund Y. Lam

**Affiliations:** 1 Department of Electrical and Electronic Engineering, The University of Hong Kong, Pokfulam, Hong Kong SAR China; 2 Department of Food Science and Nutrition, The Hong Kong Polytechnic University, Hung Hom, Hong Kong SAR China; 3 Key Lab of Education Ministry for Power Machinery and Engineering, School of Mechanical Engineering, Shanghai Jiao Tong University, 800 Dongchuan Road, Shanghai, 200240 China; 4 State Key Laboratory of Marine Pollution, City University of Hong Kong, Kowloon Tong, Hong Kong SAR China

**Keywords:** Imaging and sensing, Electrical and electronic engineering

## Abstract

Microplastic (MP) pollution has emerged as a global environmental concern due to its ubiquity and harmful impacts on ecosystems and human health. MP assessment has therefore become increasingly necessary and common in environmental and experimental samples. Microscopy and spectroscopy are widely employed for the physical and chemical characterization of MPs. However, these analytical methods often require time-consuming pretreatments of samples or expensive instrumentation. In this work, we develop a portable and cost-effective polarization holographic imaging system that prominently incorporates deep learning techniques, enabling efficient, high-throughput detection and dynamic analysis of MPs in aqueous environments. The integration enhances the identification and classification of MPs, eliminating the need for extensive sample preparation. The system simultaneously captures holographic interference patterns and polarization states, allowing for multimodal information acquisition to facilitate rapid MP detection. The characteristics of light waves are registered, and birefringence features are leveraged to classify the material composition and structures of MPs. Furthermore, the system automates real-time counting and morphological measurements of various materials, including MP sheets and additional natural substances. This innovative approach significantly improves the dynamic monitoring of MPs and provides valuable information for their effective filtration and management.

## Introduction

Microplastics (MPs) are small plastic particles with a size typically less than 5mm^1,2^. They originate from various sources such as fragmentation of larger plastic debris, shedding of microfibers from synthetic textiles during washing, and utilization of microbeads in personal care products^3^. These minute particles are ubiquitous in the environment and have been detected in diverse ecosystems, including oceans, rivers, soil, and even air^4,5^. Due to their small size and persistence, MPs pose a significant threat to the environment, wildlife, and potentially human health, as they can transfer through the food chain and serve as carriers for harmful pollutants, such as heavy metals and persistent organic pollutants^6,7^. Furthermore, the presence of MPs in aquatic systems can negatively affect the growth, reproduction, and survival of aquatic organisms, causing disruptions in ecosystems and potentially leading to a decline in biodiversity^8^. The increasing prevalence of MPs has raised global concerns, prompting calls for more sustainable practices in plastic production, consumption, and waste management^9,10^.

Identifying MPs and assessing water quality have gained increasing public attention^11^. Chemical and thermal analysis of MPs are the most commonly adopted methods in the laboratory. For instance, Raman spectroscopy can distinguish different types of MPs from the Raman spectra of the samples based on the interaction of light with the chemical bonds of a substance^12^. Microscopic techniques are also widely utilized for the physical characterization of MPs, providing detailed structural information for identification purposes^13^. These techniques include stereo, fluorescence, scanning electron, transmission electron, and atomic force microscopy, each with varied imaging capabilities suited to particular applications^14^. However, while most of the existing analytical methods are advantageous in characterizing MPs in the laboratory, they are generally unsuitable for in in situ detection and high-throughput monitoring of MPs suspended in natural water, as they cannot readily track changes in these particles^15^. Furthermore, these typical methods often require either complex and expensive facilities, or labor-intensive and time-consuming pretreatments of samples, such as Nile red staining for fluorescent imaging^16^. To avoid extensive sample preparation in the laboratory, portable devices or technologies need to be developed for fieldwork, and the imaging system should be capable of distinguishing MPs in the aqueous phase, where most of them are found.

The increasing concern for MP pollution has prompted the development of advanced techniques to effectively detect and quantify these harmful particles in various environments^17,18^. Digital holographic imaging is a versatile method that encodes the wavefront information of objects into interference patterns, facilitating rapid and precise identification of MP particles^19–21^. The interference pattern, also known as a digital hologram, is generated through a process that involves the interaction of two coherent light waves: the reference wave and the object wave^22,23^. Upon recombination, the light waves create a digital hologram that encodes both the amplitude and phase information of the scattered light at any adjustable depth, providing a comprehensive characterization of the MP samples. Subsequently, the recorded digital hologram is processed using numerical algorithms to reconstruct the complex optical field of the scattered light. Furthermore, digital holographic imaging offers a label-free, non-destructive, non-invasive approach that eliminates the need for extensive laboratory sample preprocessing^24^. This technique also offers a compact and cost-effective solution by utilizing fewer optical components, rendering it an ideal choice for field-portable applications, especially for in situ environmental monitoring^25,26^.

Polarization imaging capitalizes on the unique optical properties of MPs, which interact differently with polarized light compared to other natural materials^27,28^. By analyzing the changes in the polarization state of light upon interaction with MP particles, this technique enables the selective identification and differentiation of MPs from other particulate matter based on their distinguishable optical anisotropy of MPs^29,30^. Therefore, combining polarization and holographic imaging results in the formation of a polarization-sensitive digital hologram, which holds promise for achieving multimodal information acquisition from MPs, including morphological, optical, and chemical features. Moreover, integrated polarization holographic imaging has a relatively simple optical configuration and requires fewer sample preparation procedures, making it ideally suitable as a field-portable system that can realize in situ environmental monitoring. With the addition of a fluidic channel design, it is possible to perform high-throughput analysis of MPs in various scenarios, including aqueous environments. Concurrently, computational methods are gradually being incorporated into MP assessment, promoting rapid and accurate approaches. Zhu et al. and Huang et al. are also integrating polarization-related computational imaging techniques with more compact configurations^31–34^. In their works, they consider several complex natural environments, such as turbid water. However, the samples are still undergoing assessment, and high-throughput, efficient detection has not yet been achieved.

In addition to these advancements, deep learning (DL)-based models, such as Mask R-CNN and U-Net, have been used in the analysis of MPs^35,36^. Compared with traditional visual inspection and counting, DL-based models facilitate feature extraction to achieve automated and intelligent MP identification and quantification. Researchers have applied sophisticated DL models capable of analyzing large volumes of data, such as images and spectroscopic information, to accurately identify and quantify MP particles in various environmental samples^37,38^. DL models have shown remarkable success in recognizing complex patterns and features within holographic interference fringes, enabling rapid and reliable detection of MPs^28,39,40^. The application of artificial intelligence for multidimensional MP assessment is an emerging field.

In this work, we present a novel approach that emphasizes high-throughput detection and DL models for the dynamic monitoring of MPs. Our method employs a fluidic channel to enable in situ detection of flowing MPs and utilizes polarized digital holography to record and extract multimodal information, such as light intensity, complex holographic patterns, and polarization state, capturing the characteristics of the light wave. By leveraging birefringence features, we can classify material composition and structures. The integration of DL models automates real-time counting, morphological quantification, and classification of different plastic sheets based on large quantities of data. This comprehensive system significantly improves the dynamic monitoring of MPs, providing valuable information for their efficient filtration and management.

## Results

### Underwater MP imaging and scattering removal

**Figure 1:**
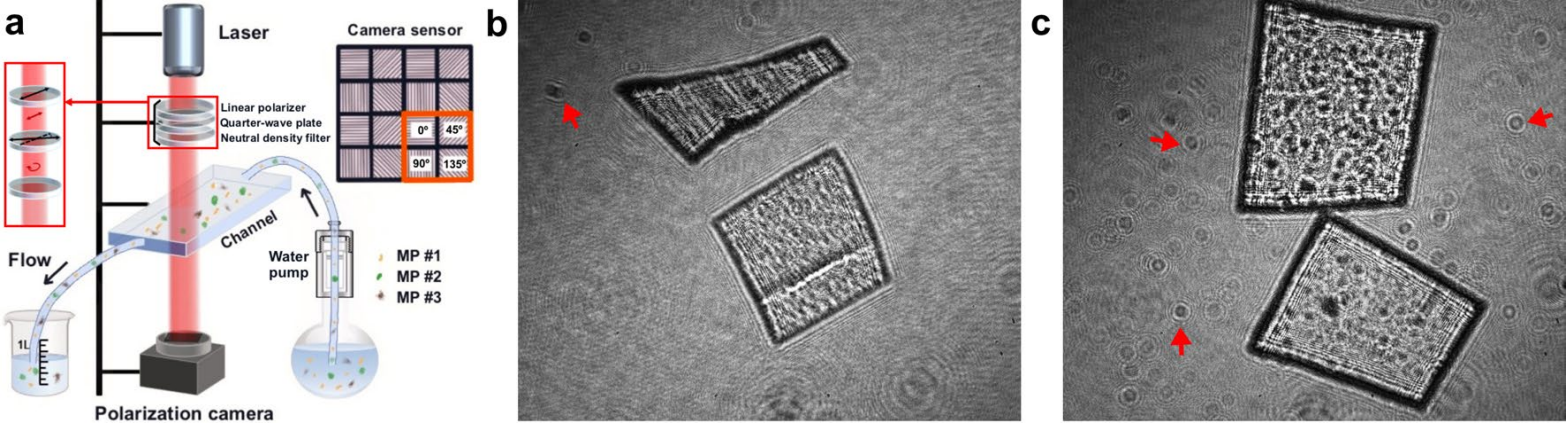
(**a**) Layout of the polarization holographic imaging system. (**b**) Intensity hologram of MP sheets in the air. (**c**) Intensity hologram of MP sheets in the water. The representative disturbance for imaging in (**b**) and (**c**) are indicated by arrows. Scale bar: 1mm.

The investigation of MPs typically involves a multi-stage process in which the particles are gathered using sieves, processed, and ultimately positioned in Petri dishes^41,42^. Furthermore, the examination and analysis of static MP samples is a labor-intensive process, frequently involving a significant degree of reliance on the operator, as it heavily depends on visual inspection. In contrast, imaging dynamic MPs offers a more efficient method for assessment compared to the time-consuming static MP analysis^41^. However, the light scattering and absorption of suspending particles often result in a veiling effect for underwater imaging, reducing the contrast on holographic fringes^43^. To address this issue, we demonstrate a compact polarization holographic device that can analyze MP samples dispersed in water and flowing through a customized glass fluidic channel, as shown in Fig. 1.

The fluidic channel is made of clear quartz glass with 95% light transmission and has dimensions of 5mm (inner thickness), 10 mm (width), and 50mm (length). This design prevents any potential disruption to the sample’s holographic fringes and is suitable for samples of various sizes. The detailed configuration of the device is illustrated in Fig. 1a. A laser source with a continuous wave and 632.8nm wavelength is used, passing through a linear polarizer (LP) and a quarter-wave plate (QWP) to generate circularly polarized light. A neutral density filter (NDF) is placed to ensure a suitable light intensity.

Polarization holographic imaging is a non-invasive technique that requires no sample preparation and offers single-shot evaluations. This characteristic enables the dynamic collection of data from flowing samples. A polarization camera is employed to simultaneously record holograms with four different polarization states at ${0}^{\circ }$, ${45}^{\circ }$, ${90}^{\circ }$, and ${135}^{\circ }$, respectively. By acquiring images at four different angles, we can effectively measure all four Stokes parameters, enabling a comprehensive characterization of the polarization state of the scattered light from the MP samples^44^. These polarization states are used to determine the sample optical anisotropy and calculate birefringence-related features (i.e. phase retardance, angle of polarization). Additionally, this system registers the holographic interference patterns between the light passing through the object and a plane reference wave. As illustrated in Fig. 1a, the laser beam is collimated by a convex lens and circularly polarized by a linear polarizer and a quarter wave plate. The fast axis of the quarter wave plate is oriented at $45^{\circ }$ with respect to that of the polarizer.

The device is designed to submerge the sample in water, which eliminates significant light reflection and scattering issues and aids in real-time classification. The intensity holograms of MP sheets in the air or aquatic environment are shown in Fig. 1b and c. Both samples perform distinguishable holographic patterns, and they can be clearly photographed with the fluidic channel. Although water may result in a disturbance compared with the air environment, it does not significantly drop the recognition performance of the patterns. Fully submerging the sample in water could reduce the interference of surface reflection.

### Real-time counting and morphological analysis

In the experimental setup, the flow rate of water is crucial for accurate fringe recording and sample movement. A water pump is applied to precisely control the speed of water flow. If the system has a high flow rate, it becomes challenging to record fringes precisely. On the other hand, if the system has a low flow rate, the samples struggle to move forward. The volume flow rate of water is optimized at 8 ml/min, allowing for stable and high-throughput detection. The device features a microfluidic channel that is capable of reaching a high throughput of 480 mL/h with accurate images. This design enables efficient MP detection in the aqueous environment.

**Figure 2:**
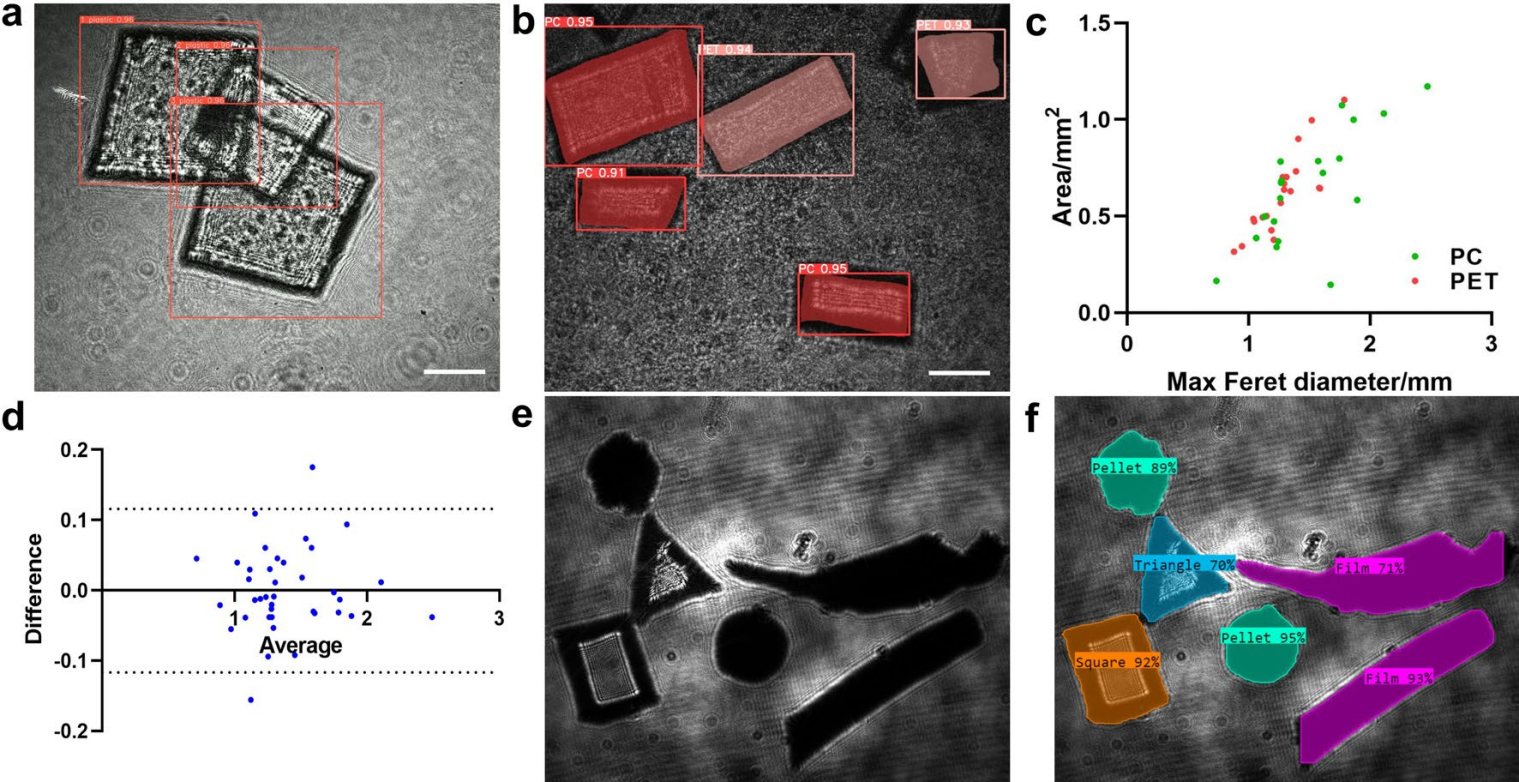
(**a**) Real-time MP counting with YOLO v5 and Strong-SORT. (**b**) Generated masks for two kinds of MPs, PC and PET. (**c**) Morphological measurement of Max Feret diameter (MFD) and area for PC and PET. (**d**) The Bland-Altman analysis comparing the MFD measurements obtained by the estimation and reference values (n = 39); the dashed lines represent the 95% limits of agreement (LOAs). (**e**) Mixed MP samples with random shapes and varied degrees of transparency. (**f**) Classification results of varied shapes shown in (**e**). Scale bar: 1mm.

The optimization ensures that samples of various shapes and materials can be effectively analyzed as they flow through the experimental device. For real-time tracking, the YOLO v5 model, a lightweight convolutional neural network (CNN), is combined with a Strong-SORT network to segment, measure, and count the MP samples^45–47^. A total of 1000 images are captured using the polarization holographic setup, as well as videos with continuous image recording. Each frame of the video is a single hologram, representing the interference pattern resulting from the superposition of object and reference waves. To further support the analysis process, the captured 1000-image dataset is divided into training-validation-test segments with a ratio of 8:1:1. This division of data facilitates the effective training of the DL model and enables reliable evaluation of its performance. As a result, the samples are counted and classified with an accuracy of up to 96$\%$. As illustrated in Fig. 2a, the number of MP samples during a certain time period can be real-time counted.

MP morphological analysis is essential for understanding the distribution, characteristics, and potential impacts of these tiny plastic particles on the environment and human health^48^. Individual masks are generated for the identified MP sheets, as shown in Fig. 2b. The Maximum Feret diameter (MFD, length) and area are employed to measure the samples’ morphology and provide valuable information for MP management (Fig. 2c). The Feret diameter is a measure of an object’s size along a specified direction and can be defined as the distance between the two parallel planes restricting the object perpendicular to that direction^49^. The MFD represents the longest dimension of the samples, and alongside the area, these elements play a crucial role in determining the appropriate filter size for MP filtration. The Bland-Altman method is commonly used to evaluate the agreement between a new tool and the reference standard^50^. Therefore, the Bland-Altman plot shown in Fig. 2d is employed to assess the consistency of MFD estimation and reference values (n = 39). The majority of data points fall within the 95% limits of agreement (LOAs), demonstrating a standard deviation (SD) of bias equal to 0.05935. Furthermore, the manually cut MP samples are mixed with MP samples collected from an in situ environment, and their shapes are accurately identified based on the generated masks, as shown in Fig. 2e and f. Both transparent and opaque samples are combined for shape recognition. In addition, note that our current system is primarily used to analyze transparent and translucent samples, and can distinguish samples of different shapes. The shapes of MP samples mainly include fragments, pellets, fibers, films, foam, and beads^51^.

To compensate for the limited spatial resolution of this imaging system, a particle analyzer is used to detect micron-size MP samples with more screening parameters^52^. The particle analyzer is used to analyze ground MPs, enabling high-throughput analysis of the placed samples. As shown in Table 1, micron-scale MP particles are analyzed in multiple dimensions and the mean values are listed as reference data. Circularity measures how closely a shape resembles a perfect circle, which has a circularity value of 1, whereas a highly elongated object approaches a value of 0. The circularity of MPs reflects their interaction with the environment^53^.

**Table 1:** Morphological analysis of CryoMill ground MP particles using particle analyzer Morphologi.

	Particles	Circularity	SE volume/μm^3^	Length/μm	Width/μm	Max diameter/μm
PC	1515	0.856	1.69e^6^	57.41	34.97	921.59
PET	2899	0.901	7.42e^5^	36.62	24.01	560.44
PP	2949	0.899	2.78e^5^	20.38	12.55	569.44
PS	2559	0.883	3.69e^5^	43.72	28.62	402.35
PVC	930	0.854	1.14e^6^	58.67	37.49	550.52

### Material classification by polarization holographic features

In this study, we analyze samples that included six types of transparent and colorless plastic materials commonly used in consumer goods production: polycarbonate (PC), polyethylene terephthalate (PET), polyvinyl chloride (PVC), polypropylene (PP), polystyrene (PS), and polymethyl methacrylate (PMMA). These samples are manually cut into small fragments with a size below 5mm and are difficult to distinguish with the naked eye. Additional materials such as glass, lens paper, and algae are included in the control group to enrich sample diversity. Glass, typically an amorphous solid with no long-range crystal structure, is not birefringent under normal conditions, making it a suitable reference^54^. Meanwhile, different plastic products exhibit varying degrees of birefringence, which allows for material distinction.

**Figure 3:**
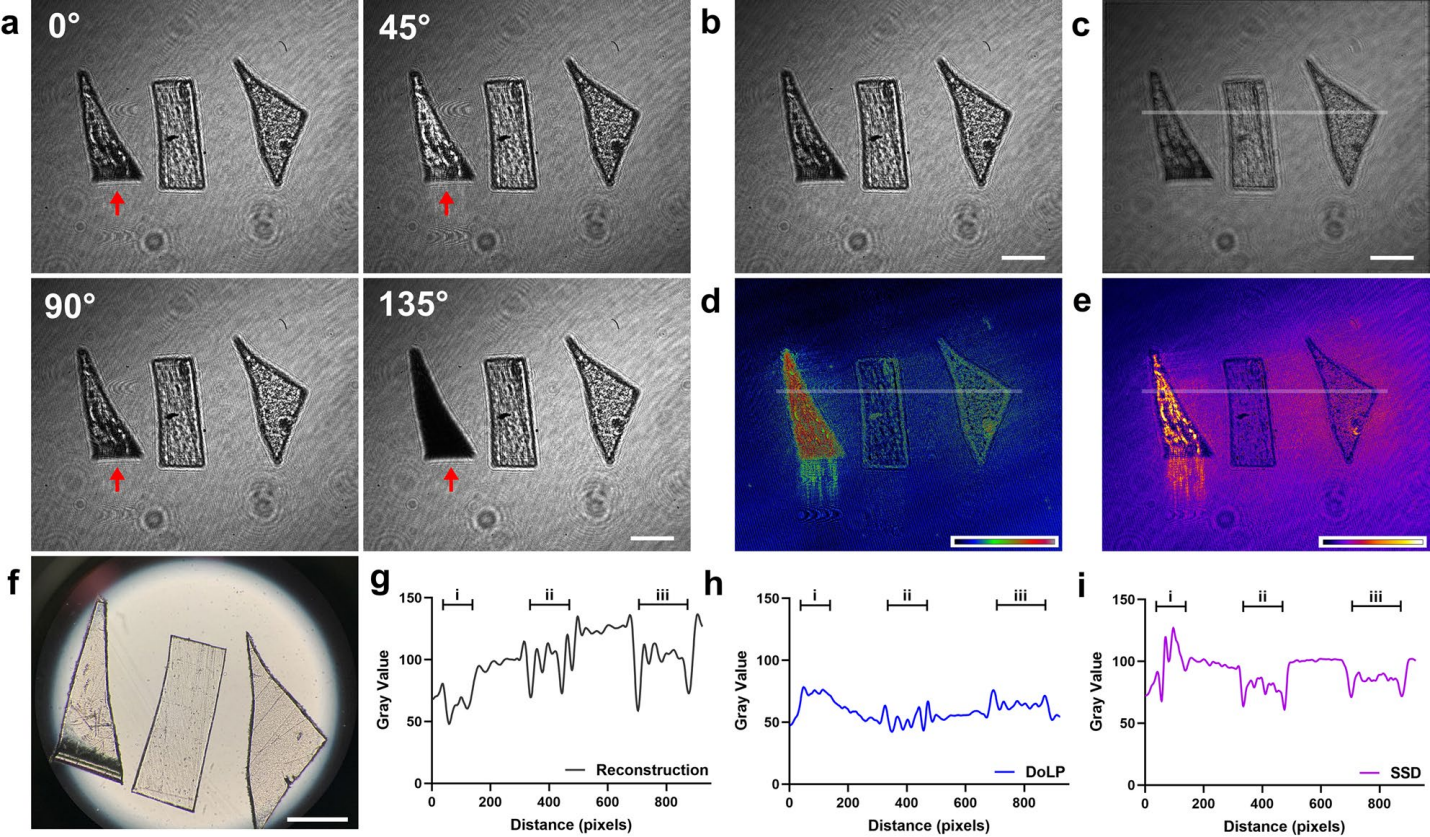
(**a**) Representative polarized digital holograms at four angles. (**b**) Processed intensity holograms of identical samples. (**c**) Holographic reconstruction of (**b**). (**d**) Degree of linear polarization (DoLP) features of MP sheets. (**e**) The stack standard deviation (SSD) image illustrates the birefringence-related features. (**f**) Microscope image of MP sheets. (**g**) Intensity value distribution derived from (**c**). (**h**) DoLP value distribution obtained from (**d**). (**i**) SSD value distribution extracted from (**e**). Scale bar: 1mm.

The samples are imaged by a stereo microscope and compared with polarized holograms. The intensity differences in the four-angle images, as shown in Fig. 3a, indicate their optical anisotropy, which is helpful for material discrimination. The intensity of the MP sheet i with strong birefringence as indicated by the arrows changes significantly in four images compared with the other two MP sheets, ii and iii. Holographic fringes of different patterns are displayed in Fig. 3b. As the complex light field is encoded in the holograms, our imaging system can numerically reconstruct them, as shown in Fig. 3c. The reconstructed image exhibits a minor distinction from the original holograms in terms of morphological measurements and material identification. To facilitate real-time MP tracking and efficient measurement, the numerical reconstruction is omitted in subsequent experiments.

MP material classification plays a crucial role in understanding the distribution and potential impacts of these contaminants on the environment. Some plastic materials are optically anisotropic and exhibit birefringence^55^. Birefringence refers to the optical characteristic of a material having a refractive index that depends on the polarization and propagation direction of light^56^. The six kinds of samples are all transparent polymers but vary in birefringence. Plastic sheets with anisotropy can alter the polarization state of transmitted light at different angles, and also influence the polarization parameters such as the degree of linear polarization (DoLP) as shown in Fig. 3d. Moreover, the MP sheets with higher anisotropy show significant intensity changes at different angles, consistent with their respective birefringence values. The four images are quantified as total input, and the stack standard deviation (SSD) is used to demonstrate the birefringence difference for further training and identification, as presented in Fig. 3e. Meanwhile, Fig. 3f presents a stereo microscope image of six samples, which are difficult to distinguish when they are transparent. Additionally, line profiles of the holographic reconstruction, DoLP, and SSD are measured accordingly, demonstrating that DoLP and SSD measurements make it easier to distinguish materials with birefringence (Fig. 3g–i).

**Figure 4:**
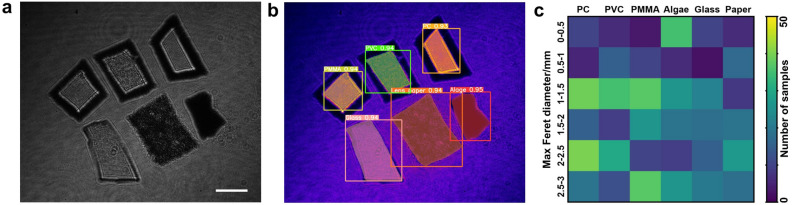
(**a**) Intensity images of six types of MP samples. (**b**) Sample identification results based on polarization holographic features. (**c**) Heatmap indicates the number of samples in different sizes and material groups. Scale bar: 1mm.

By utilizing polarization holographic imaging, we extract unique features from the MP samples and use them to train the DL model for classification purposes. The images of six types of samples are shown in Fig. 4a and further classified, corresponding to the SSD in Fig. 4b. This classification helps assess the fate and transport of MPs in the environment. Furthermore, determining the material composition enables the development of targeted management strategies to reduce the release of specific MP types, ultimately mitigating their environmental impacts. A comprehensive heatmap is generated to visualize the distribution of MPs in the samples, illustrating the number of MPs in different size ranges and material types, as shown in Fig. 4c. This heatmap provides a clear overview of the MP distribution and allows for a better understanding of the filtration requirements for different materials and sizes.

### Comparison of results with Raman spectra

A single imaging method often proves insufficient for accurately and reliably identifying MPs, as they can vary widely in size, shape, and polymer composition, particularly when embedded in complex environmental matrices. To overcome these limitations, a combination of two or more analytical techniques is often employed^57^. Typically, physical characterization is coupled with chemical characterization, such as spectroscopy, to produce a comprehensive analysis^58^.

**Figure 5:**
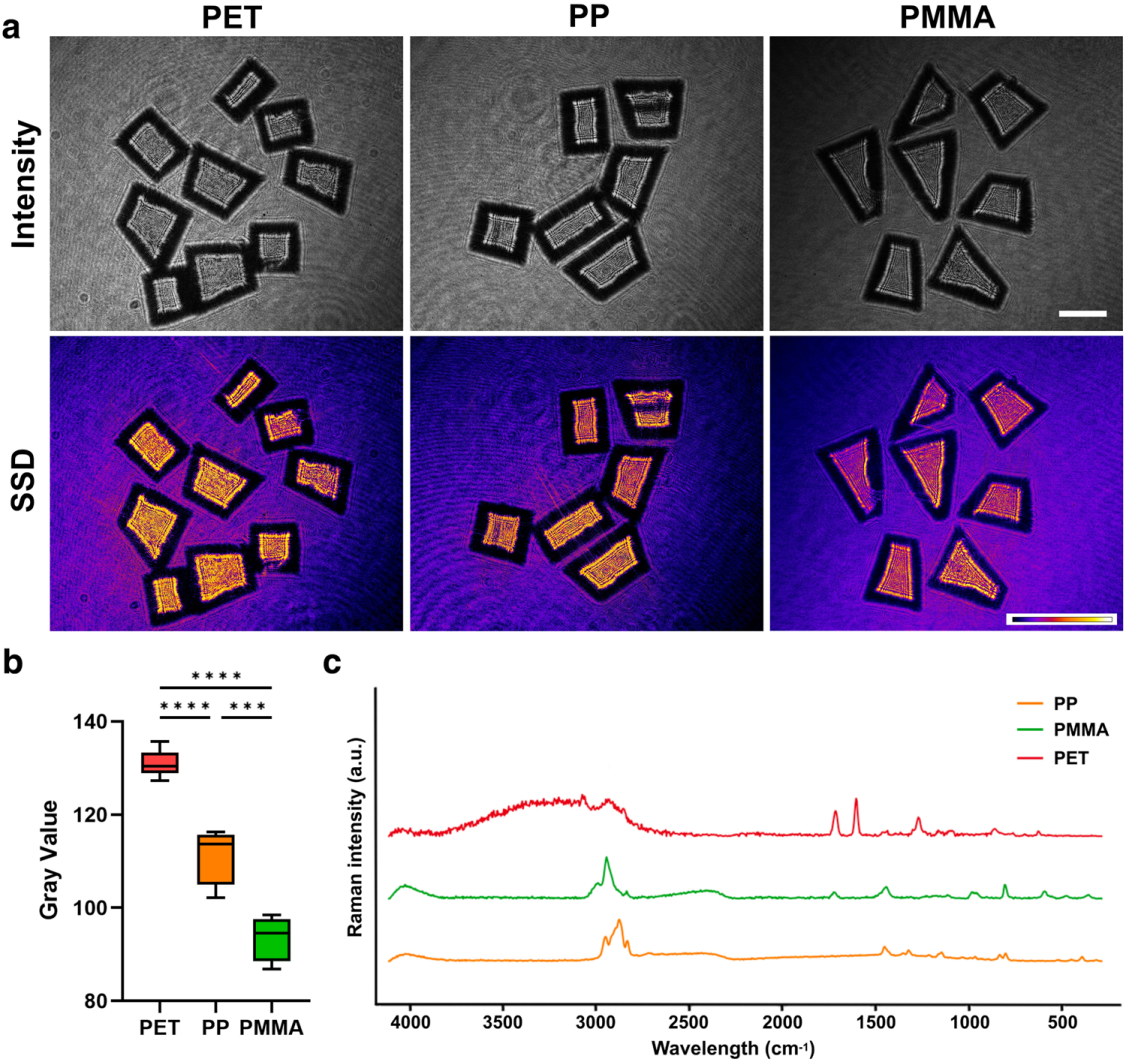
(**a**) Representative MP sheets and their imaging features. (**b**) The mean value distribution of measured SSD groups, displaying statistically significant differences (n=3). (**c**) Raman spectra of representative MP sheets. Scale bar: 1mm.

Raman spectroscopy imaging has proven to be particularly valuable in the study of MPs, as it allows for the accurate identification and classification of various polymer types based on their unique spectral signatures and molecular composition. Raman spectroscopy has been considered the ground truth for MP detection, as it provides the identification of multiple characteristic peaks^59^. These peaks represent the current state of the atomic structure and Raman reaction and can be used as a reference for polarization holographic image features.

We compare the identified MP material results with their corresponding Raman spectra, as shown in Fig. 5. The intensity images for three distinct types of MPs and their corresponding SSD feature images effectively demonstrate the variations in birefringence among them (Fig. 5a). Figure 5b shows the mean value distribution of measured SSD groups for three representative types of MP samples. The related spectra are consistent with each other, which further supports the conclusions about correct identification (Fig. 5c). Moreover, further investigation is required to fully understand the mapping relationship between the polarization holographic patterns and the Raman spectrum features.

## Discussion

In this study, we successfully demonstrate a novel approach to assess MP morphological parameters and material types based on polarization holographic imaging features using DL models. The results obtained from our experiments and analyses show the effectiveness and potential of this method in identifying and categorizing different types of MP materials. Our research introduces a real-time, high-throughput MP analyzer that leverages multidimensional information to enhance the quantification of MPs and provide a reference for treatment strategies. Moreover, the high classification accuracy, robustness, and generalizability of this approach make it a promising tool for future research and practical applications in assessing and managing MP pollution.

MP pollutants have become a global environmental concern, leading to the development and application of various detection methods, including imaging-based, thermal, and chemical analysis techniques^58^. Practical MP detection often requires the use of multiple methods to achieve reliable results. Combining thermal and chemical techniques with imaging-based methods can provide a more comprehensive approach to MP detection. It is highly desirable to identify and quantify MP particles together with their chemical compositions and morphological distributions. In recent years, several emerging imaging techniques have been applied to the field of MP detection. These imaging techniques can provide valuable information for MP identification and analysis and potentially lead to new insights into the distribution and impact of MPs in the environment. For example, a combination of neutron and X-ray tomography has been tested to detect MP particles in sandy sediments^60^. Moreover, flow cytometry (FlowCam) can be used to detect and analyze MPs in water samples^61,62^, combining high-resolution imaging with flow cytometry to capture images of particles. As more technologies are developed, additional MP features can be acquired. We can further explore the possibility of enhancing the integration of new advanced technologies and the current detection methods for MPs.

However, there are still complex situations for MP analysis. For instance, multiple scattering and absorption exist in real seawater samples that are usually mixed with a lot of impurities, affecting the imaging effect^63^. Tracking and detecting MPs in cases of complicated turbid water scattering is our next focus of work^64,65^. It has been primarily demonstrated that our imaging system is capable of seeing through scattering media and obtaining multimodal information about the object. The results suggest that polarization features can substantially improve image contrast even in highly turbid water^31^. In addition, an objective lens will be introduced into the system to increase the detection of full-scale samples, such as those on the micrometer scale. Simultaneously, more natural samples will be incorporated to make the results closer to the seawater situation, making the detection results of the system more practical.

## Methods

### Experimental setup, calibration and data collection

In this study, polarization holographic imaging technology is applied for feature capture and fieldwork using a compactly designed system. A monochromatic laser light source with a wavelength of 632.8 nm is employed. An LP and a QWP are combined to generate a circularly polarized laser beam. An NDF is placed to ensure the light intensity is suitable. The uniformly distributed circularly polarized light then passed through the sample plate and interacted with the samples, encoding the sample feature information with wavefront deformation and polarization state modification. A polarization CMOS camera (Crevis Tech, MC-A500P-163) is used to record polarization-sensitive digital holograms with four different polarization states. The captured image has a resolution of 2464 pixels $\times$2056 pixels. The pixels with size 3.45$\upmu \!{\textrm{m}}$ are arranged in a group of $2\times 2$ grid arrays, which simultaneously capture the polarization states at ${0}^{\circ }$, ${45}^{\circ }$, ${90}^{\circ }$, and ${135}^{\circ }$, respectively. These polarization states are used to calculate birefringence-related features.

To ensure the accuracy and reliability of the system, a calibration process has been implemented. This system calibration encompasses circularly polarized light calibration and lateral resolution calibration, accounting for the impacts of the medium, the sample, and dispersion. A reference sample, such as glass without birefringence, is utilized to calibrate the polarization holographic imaging system. The light intensity distribution at four distinct angles is adjusted to achieve a uniformly circularly polarized light beam, and a non-birefringent glass sample is employed to validate the DoLP parameter. Reference samples are prepared with known physical parameters, including size and shape, under consistent medium and dispersion conditions. Analyzing the reference sample enables the determination of the relationship between the measured optical phase and the established physical parameters. In this system, the calibrated spatial resolution is 20μm. A USAF-1951 target (Edmund Optics, USA) is used for resolution characterization. The examination of the reference sample allows for the estimation of potential errors and uncertainties in the measurements, thereby ensuring the reliability of the obtained results.

Sample particle detection in flow is performed using a customized fluidic channel made of 95% light transmission quartz glass. The dimensions of the channel are designed according to the MP size range: 5 mm (inner thickness), 10 mm (width), and 50 mm (length). The flow speed is precisely controlled by a water pump (Kamoer Tech, NKCP-C-SO4B), with a speed control range starting from 2 ml/min and capable of reaching a maximum volume flow rate of 15 ml/min. The optimal speed should be determined according to the sample situation. In this study, the optimized speed is set at 8 ml/min for stable MP movement.

### Generation of digital holograms and reconstrunction

In a typical digital holography system, the formation of the holographic fringe pattern is achieved by the spatial superposition of the complex reference wave ($U_R$) and object wave ($U_O$). $U_R$ and $U_O$ are initially plane waves. A wavefront deformation is introduced in $U_O$ with the reflection, transmission, and scattering from the object. A photodetector such as a charge-coupled device (CCD) or complementary metal-oxide-semiconductor (CMOS) camera is used to record the intensity distribution (*I*(*x*, *y*)) of the resulting holographic patterns.

1 $$\begin{aligned} I=\left| U_R + U_O \right| ^{2} = \left| U_R \right| ^2 + \left| U_O \right| ^2 + U_O^*U_R + U_OU_R^*, \end{aligned}$$

where ^∗^ denotes the complex conjugate of the referring part. The spatial coordinates (*x*, *y*) are omitted in the equation for clarity. The first two parts of the result are the DC-terms and acting as noise. DC-terms can be limited by applying a numerical filter in the frequency domain or subtracting the mean intensity values. The third and fourth terms are the generated interference patterns. The last term will be considered for further modulation and reconstruction.

Numerical reconstruction of holograms is employed to decode the complex wavefront encoded in the captured holograms, revealing the distributions of amplitude and phase at any desired depth. This process facilitates the extraction of valuable information about the sample, including its morphological characteristics. The angular spectrum function, a widely used method, is applied to calculate the forward or backward propagation of light, allowing for the reconstruction of images across various focal planes. This function is mathematically expressed as

2 $$\begin{aligned} \Gamma (x, y, z)=\mathscr {F}^{-1}\left\{ \mathscr {F}\left[ E_{R}^{*}(x, y) h(x, y)\right] \times \exp \left( -i \frac{2 \pi z}{\lambda } \sqrt{1-\lambda ^{2} f_{x}^{2}-\lambda ^{2} f_{y}^{2}}\right) \right\} , \end{aligned}$$

where $\Gamma (x, y, z)$ represents the reconstructed complex wavefront with coordinates (*x*, *y*, *z*), $\mathscr {F}$ denotes the Fourier transform, $E_{R}^{*}$ is the complex conjugate of the reference wave, *h* is the captured hologram, $\lambda$ is the wavelength of illumination light, and $f_{x}$ and $f_{y}$ are the transverse spatial frequencies.

### Polarization holographic feature analysis

Holograms at four different angles are simultaneously recorded to obtain a comprehensive characterization of the polarization states of the MP samples. The intensity differences in the four-angle images indicate their optical anisotropy, which is beneficial for material classification. To facilitate material classification, the SSD of four polarized angle images is calculated using Matlab software (Mathworks, US) to facilitate material classification.

By acquiring images at four distinct angles, we can effectively measure Stokes parameters, enabling a complete analysis of the polarization state. Moreover, the polarization states of light are characterized using Linear Stokes parameters

3 $$\begin{aligned} I(x,y)&=I^{0}(x,y)+I^{90}(x,y), \end{aligned}$$

4 $$\begin{aligned} Q(x,y)&=I^{0}(x,y)-I^{90}(x,y), \end{aligned}$$

5 $$\begin{aligned} U(x,y)&=I^{45}(x,y)-I^{135}(x,y), \end{aligned}$$

where *I* represents the grayscale intensity of a hologram at a certain polarization angle.

Moreover, specific polarization parameters are derived from the captured holographic images based on Stokes parameters to characterize the polarization response of MP sheet samples, such as the degree of linear polarization (DoLP $\in [0,1]$)

6 $$\begin{aligned} \textrm{DoLP}(x,y)&=\frac{\sqrt{Q^{2}(x, y)+U^{2}(x, y)}}{I(x, y)}, \end{aligned}$$

and the angle of polarization (AoP $\in [-\frac{\pi }{4},+\frac{\pi }{4}]$)

7 $$\begin{aligned} \textrm{AoP}(x,y)&=\frac{1}{2} \tan ^{-1} \frac{U(x, y)}{Q(x, y)}. \end{aligned}$$

The DoLP represents the magnitude of the linear polarization state, while the AoP indicates the orientation of the polarization state. Furthermore, both DoLP and AoP are independent of particle geometrical parameters, such as shape, area, size, and perimeter, and remain consistent across a wide range of reconstruction distances. This information is crucial for the reliable identification and quantification of MPs.

### Sample preparation and image processing

In this study, six different types of MP sheets are applied: PC, PET, PVC, PP, PS, and PMMA. These samples with varying birefringence values are purchased from Xinsheng Plastic Material Company, China. To ensure sample diversity, additional materials such as glass, lens paper, and algae are included in the control group.

The samples are imaged using a stereo microscope (Nikon Model Eclipse Ni-U, Japan) with a digital camera. The captured MP images by the polarization CMOS camera are accurately labeled using ImageJ software (National Institutes of Health, US), and the dataset is divided into training, validation, and test sets at a ratio of 8:1:1. The YOLO v5 model, a lightweight CNN, is combined with a Strong-SORT network to segment, measure, and count the MP samples. The DL model is further fine-tuned. To fine-tune the hyperparameters, specifically the learning rate, batch size, and whether augmentations should be used, a manual grid search is performed. The area and MFD values are calculated by a self-developed Python script. The scatter diagram and line chart are visualized by GraphPad Prism software (GraphPad Inc, USA).

### Reference data acquisition

MP particles with an approximate size of 100μm in size, are further processed using a Retsch CryoMill (Haan Tech, Germany). The ultra-low operating temperature is maintained by circulating liquid nitrogen around the grinding chamber, which is made of zirconium oxide. Subsequently, the ground MP particles are analyzed for various morphological parameters using a particle analyzer Morphologi 4 (Malvern Panalytical, UK), including circularity, volume, length, width, and diameter.

Raman spectra serve as a reference for MP identification. MP sheets are dried at 40 °C and the Raman spectra are assessed using a Optical-Photothermal InfraRed mIRage (California, USA), by illuminating the sample with a mid-IR pulsed tunable laser of 532nm detection laser for 10 s exposure time. Raman spectra of the MP samples are acquired in the wavenumber range of 200–4170 cm^-1^. Baseline correction and smoothing of the acquired spectra are performed with the PTIR Studio 4.4 software.

## Data Availability

The datasets used and/or analysed during the current study available from the corresponding author on reasonable request.

## References

[1] Akdogan Z, Guven B (2019). Microplastics in the environment: A critical review of current understanding and identification of future research needs. Environ. Pollut..

[2] Thompson RC (2004). Lost at sea: Where is all the plastic?. Science.

[3] Koelmans AA (2022). Risk assessment of microplastic particles. Nat. Rev. Mater..

[4] Rillig MC, Lehmann A (2020). Microplastic in terrestrial ecosystems. Science.

[5] Sharma S, Chatterjee S (2017). Microplastic pollution, a threat to marine ecosystem and human health: A short review. Environ. Sci. Pollut. Res..

[6] Vethaak AD, Legler J (2021). Microplastics and human health. Science.

[7] Kinigopoulou V, Pashalidis I, Kalderis D, Anastopoulos I (2022). Microplastics as carriers of inorganic and organic contaminants in the environment: A review of recent progress. J. Mol. Liq..

[8] Luo H (2022). Environmental behaviors of microplastics in aquatic systems: A systematic review on degradation, adsorption, toxicity and biofilm under aging conditions. J. Hazard. Mater..

[9] Chow, C.-F., So, W.-M. W., Cheung, T.-Y. & Yeung, S.-K. D. Plastic waste problem and education for plastic waste management. *Emerging Practices in Scholarship of Learning and Teaching in a Digital Era* 125–140 (2017).

[10] Shin S-K, Um N, Kim Y-J, Cho N-H, Jeon T-W (2020). New policy framework with plastic waste control plan for effective plastic waste management. Sustainability.

[11] Cowger W, Gray A (2020). Critical review of processing and classification techniques for images and spectra in microplastic research. Appl. Spectrosc..

[12] Schymanski D, Goldbeck C, Humpf HU, Furst P (2018). Analysis of microplastics in water by micro-Raman spectroscopy: Release of plastic particles from different packaging into mineral water. Water Res..

[13] Baruah A, Sharma A, Sharma S, Nagraik R (2022). An insight into different microplastic detection methods. Int. J. Environ. Sci. Technol..

[14] Mariano S, Tacconi S, Fidaleo M, Rossi M, Dini L (2021). Micro and nanoplastics identification: Classic methods and innovative detection techniques. Front. Toxicol..

[15] Li J (2023). Recognition of microplastics suspended in seawater via refractive index by mueller matrix polarimetry. Mar. Pollut. Bull..

[16] Shim WJ, Song YK, Hong SH, Jang M (2016). Identification and quantification of microplastics using nile red staining. Mar. Pollut. Bull..

[17] Priya A (2023). Removing microplastics from wastewater using leading-edge treatment technologies: A solution to microplastic pollution-a review. Bioprocess Biosyst. Eng..

[18] Fu W, Min J, Jiang W, Li Y, Zhang W (2020). Separation, characterization and identification of microplastics and nanoplastics in the environment. Sci. Total Environ..

[19] Ren Z, Xu Z, Lam EY (2019). End-to-end deep learning framework for digital holographic reconstruction. Adv. Photonics.

[20] Zhu Y, Yeung CH, Lam EY (2021). Digital holographic imaging and classification of microplastics using deep transfer learning. Appl. Opt..

[21] Zhu, Y., Yeung, C. H. & Lam, E. Y. Holographic classifier: Deep learning in digital holography for automatic micro-objects classification. in *booktitle2020 IEEE 18th International Conference on Industrial Informatics (INDIN)*, vol. 1, 515–520 (IEEE, 2020).

[22] Goodman JW (2017). Introduction to Fourier Optics.

[23] Wang Z (2022). Digital holography as metrology tool at micro-nanoscale for soft matter. Light Adv. Manuf..

[24] Merola F (2018). Searching and identifying microplastics in marine environment by digital holography. Eur. Phys. J. Plus.

[25] Asamoah BO (2021). Towards the development of portable and in situ optical devices for detection of micro-and nanoplastics in water: A review on the current status. Polymers.

[26] Schnitzler L (2021). Lensless digital holographic microscopy as an efficient method to monitor enzymatic plastic degradation. Mar. Pollut. Bull..

[27] Valentino M (2022). Intelligent polarization-sensitive holographic flow-cytometer: Towards specificity in classifying natural and microplastic fibers. Sci. Total Environ..

[28] Zhu Y, Yeung CH, Lam EY (2021). Microplastic pollution monitoring with holographic classification and deep learning. J. Phys. Photonics.

[29] Yang Y, Huang H, Guo C (2020). Polarization holographic microscope slide for birefringence imaging of anisotropic samples in microfluidics. Opt. Express.

[30] Wang J, Tan X (2022). Linear polarization holography. Opto-Electron. Sci..

[31] Huang J, Zhu Y, Li Y, Lam EY (2023). Snapshot polarization-sensitive holography for detecting microplastics in turbid water. ACS Photonics.

[32] Zhu, Y., Li, Y., Huang, J. & Lam, E. Y. Smart polarization and spectroscopic holography for real-time microplastics identification. *Commun. Eng.* (2024).

[33] Zhu, Y., Li, Y., Huang, J., Zhang, Y. & Lam, E. Y. Holographic and polarization features analysis for microplastics characterization and water monitoring. In *booktitleMultimodal Sensing and Artificial Intelligence: Technologies and Applications III in SPIE Optical Metrology*, 12621–33 (SPIE, 2023).

[34] Li, Y., Zhu, Y., Huang, J., Zhang, Y. & Lam, E. Y. Polarization holographic imaging for high-throughput microplastic analysis. In *booktitleDigital Holography and Three-Dimensional Imaging*, HM1D–6 (Optica Publishing Group, 2023).

[35] Lee KS (2022). U-net skip-connection architectures for the automated counting of microplastics. Neural Comput. Appl..

[36] Lorenzo-Navarro J (2021). Deep learning approach for automatic microplastics counting and classification. Sci. Total Environ..

[37] Brandt J, Mattsson K, Hassellov M (2021). Deep learning for reconstructing low quality FTIR and Raman spectra a case study in microplastic analyses. Anal. Chem..

[38] Zhu, Y., Yeung, C. H. & Lam, E. Y. Digital holography with deep learning and generative adversarial networks for automatic microplastics classification. In *booktitleHolography, Diffractive Optics, and Applications X*, vol. 11551, 22–27 (SPIE, 2020).

[39] Zeng T, Zhu Y, Lam EY (2021). Deep learning for digital holography: A review. Opt. Express.

[40] Zhu Y, Lo HKA, Yeung CH, Lam EY (2022). Microplastic pollution assessment with digital holography and zero-shot learning. APL Photonics.

[41] Cacace, T. *et al.* Compact holographic microscope for imaging flowing microplastics. In *booktitle2021 International Workshop on Metrology for the Sea; Learning to Measure Sea Health Parameters (MetroSea)*, 229–233 (IEEE, 2021).

[42] Prume JA, Gorka F, Löder MG (2021). From sieve to microscope: An efficient technique for sample transfer in the process of microplastics’ quantification. MethodsX.

[43] Zhu Y, Zeng T, Liu K, Ren Z, Lam EY (2021). Full scene underwater imaging with polarization and an untrained network. Opt. Express.

[44] Otani, Y., Endo, N., Hagen, N. & Shibata, S. Imaging microplastics consumed by water organisms using a full-stokes polarization camera. In *booktitleBiomedical Imaging and Sensing Conference 2021*, vol. 11925, 88–89 (SPIE, 2021).

[45] Shishkin, I. E. & Grekov, A. N. Implementation of yolov5 for detection and classification of microplastics and microorganisms in marine environment. In *booktitle2023 International Russian Smart Industry Conference (SmartIndustryCon)*, 230–235 (IEEE, 2023).

[46] Jocher, G. *et al.* ultralytics/yolov5: v7. 0-yolov5 sota realtime instance segmentation. *Zenodo* (2022).

[47] Du, Y. *et al.* Strongsort: Make deepsort great again. *IEEE Transactions on Multimedia* (2023).

[48] Helm PA (2017). Improving microplastics source apportionment: A role for microplastic morphology and taxonomy?. Anal. Methods.

[49] Li Y, Parkinson DY, Feng J, Xia C-H, Gong X (2021). Quantitative X-ray tomographic analysis reveals calcium precipitation in cataractogenesis. Sci. Rep..

[50] Bland JM, Altman DG (1995). Comparing methods of measurement: Why plotting difference against standard method is misleading. Lancet.

[51] Pizzurro F, Recchi S, Nerone E, Salini R, Barile NB (2022). Accumulation evaluation of potential microplastic particles in *Mytilus galloprovincialis* from the Goro Sacca (Adriatic sea, Italy). Microplastics.

[52] Fang JKH (2023). Adverse impacts of high-density microplastics on juvenile growth and behaviour of the endangered tri-spine horseshoe crab *Tachypleus tridentatus*. Mar. Pollut. Bull..

[53] Severini E (2022). River-groundwater interaction and recharge effects on microplastics contamination of groundwater in confined alluvial aquifers. Water.

[54] Mueller H (1935). Theory of photoelasticity in amorphous solids. Physics.

[55] Hong N, Synowicki RA, Hilfiker JN (2017). Mueller matrix characterization of flexible plastic substrates. Appl. Surf. Sci..

[56] Siebourg W (1990). Birefringence-an important property of plastic substrates for magneto-optical storage disks. Polym. Eng. Sci..

[57] Song YK (2015). A comparison of microscopic and spectroscopic identification methods for analysis of microplastics in environmental samples. Mar. Pollut. Bull..

[58] Wang Z-M, Wagner J, Ghosal S, Bedi G, Wall S (2017). SEM/EDS and optical microscopy analyses of microplastics in ocean trawl and fish guts. Sci. Total Environ..

[59] Leung MM-L (2021). Improved Raman spectroscopy-based approach to assess microplastics in seafood. Environ. Pollut..

[60] Tötzke C, Oswald SE, Hilger A, Kardjilov N (2021). Non-invasive detection and localization of microplastic particles in a sandy sediment by complementary neutron and X-ray tomography. J. Soils Sedim..

[61] Nelson, H. *et al.* Use of imaging flow cytometry (FlowCam) in the study of microplastics. In *booktitleOcean Sciences Meeting* (Oregon Convention Center Portland, 2018).

[62] Woods MN, Stack ME, Fields DM, Shaw SD, Matrai PA (2018). Microplastic fiber uptake, ingestion, and egestion rates in the blue mussel (*Mytilus edulis*). Mar. Pollut. Bull..

[63] Li H (2023). Underwater active polarization descattering based on a single polarized image. Opt. Express.

[64] Wei Y, Han P, Liu F, Shao X (2022). Estimation and removal of backscattered light with nonuniform polarization information in underwater environments. Opt. Express.

[65] Zhang Y, Chan SH, Lam EY (2023). Photon-starved snapshot holography. APL Photonics.

